# Superior mesenteric vein reconstruction during pancreatoduodenectomy using a dilated right ovarian vein in a patient at future risk for pelvic congestion syndrome: a case report

**DOI:** 10.1186/s40792-022-01421-w

**Published:** 2022-04-13

**Authors:** Yuki Takahashi, Kenichi Matsuo, Hideyuki Oyama, Ryuichi Sekine, Akihiro Nakamura, Tsuneyuki Uchida, Mikio Makuuchi, Kuniya Tanaka

**Affiliations:** 1 Department of General and Gastroenterological Surgery, Showa University Fujigaoka Hospital, 1-30 Fujigaoka, Aoba-ku, Yokohama, Kanagawa 227-8501 Japan; 2 Department of Surgery, Sannoudai Hospital, 4-1-38, Higashi-ishioka, Ishioka, Ibaraki 315-0037 Japan

**Keywords:** Pancreatoduodenectomy, Superior mesenteric vein resection and reconstruction, Pelvic congestion syndrome

## Abstract

**Background:**

Pancreatoduodenectomy including resection of the superior mesenteric vein (SMV) is increasingly performed for right-sided pancreatic ductal adenocarcinoma invading the wall of that vessel. Various venous grafts may be chosen for reconstruction. We present a woman with pancreatic cancer who underwent such a pancreatoduodenectomy with venous reconstruction using a dilated right ovarian vein.

**Case presentation:**

A 71-year-old woman with cancer involving the pancreatic head, uncinate process, and SMV underwent pancreatoduodenectomy with SMV resection. Reconstruction used a portion of the right ovarian vein that was markedly dilated and had placed her at risk for pelvic congestion syndrome (PCS). Graft patency was confirmed 8 months after surgery. She now finished receiving adjuvant chemotherapy and has no symptoms of PCS.

**Conclusion:**

If an ovarian vein has sufficient diameter, it can be used to reconstruct the resected segment of the SMV during pancreatoduodenectomy in suitable patients.

## Background

Pancreatoduodenectomy is the only curative treatment for right-sided pancreatic ductal adenocarcinoma. Complete tumor resection with negative margins is essential for potentially curative treatment. In some patients with localized pancreatic cancer, negative margins can be achieved only by including resection of a vessel such as the portal vein (PV) or superior mesenteric vein (SMV). Further, recent advances in perioperative chemotherapy for pancreatic cancer can facilitate conversion of unresectable to resectable disease. As a result, resection and reconstruction of an involved vessel during pancreatoduodenectomy has become more common. However, specific aspects of venous resection and reconstruction using venous grafts vary considerably between cases.

Millions of women worldwide experience chronic pelvic pain at some time in life, with an occurrence rate possibly approaching 40% [1]. One cause of chronic pelvic pain is vascular congestion resulting from retrograde flow in an incompetent ovarian vein, termed pelvic congestion syndrome (PCS). Symptoms of this condition in addition to pelvic pain may vary. Etiologies include both mechanical and hormonal factors contributing to venous insufficiency and dilation [2].

Here we describe a patient with cancer of the pancreatic head and uncinate process who underwent pancreatoduodenectomy with SMV resection and reconstruction using a right ovarian vein graft. In this case, an additional benefit may be prevention of PCS.

## Case presentation

**Fig. 1:**
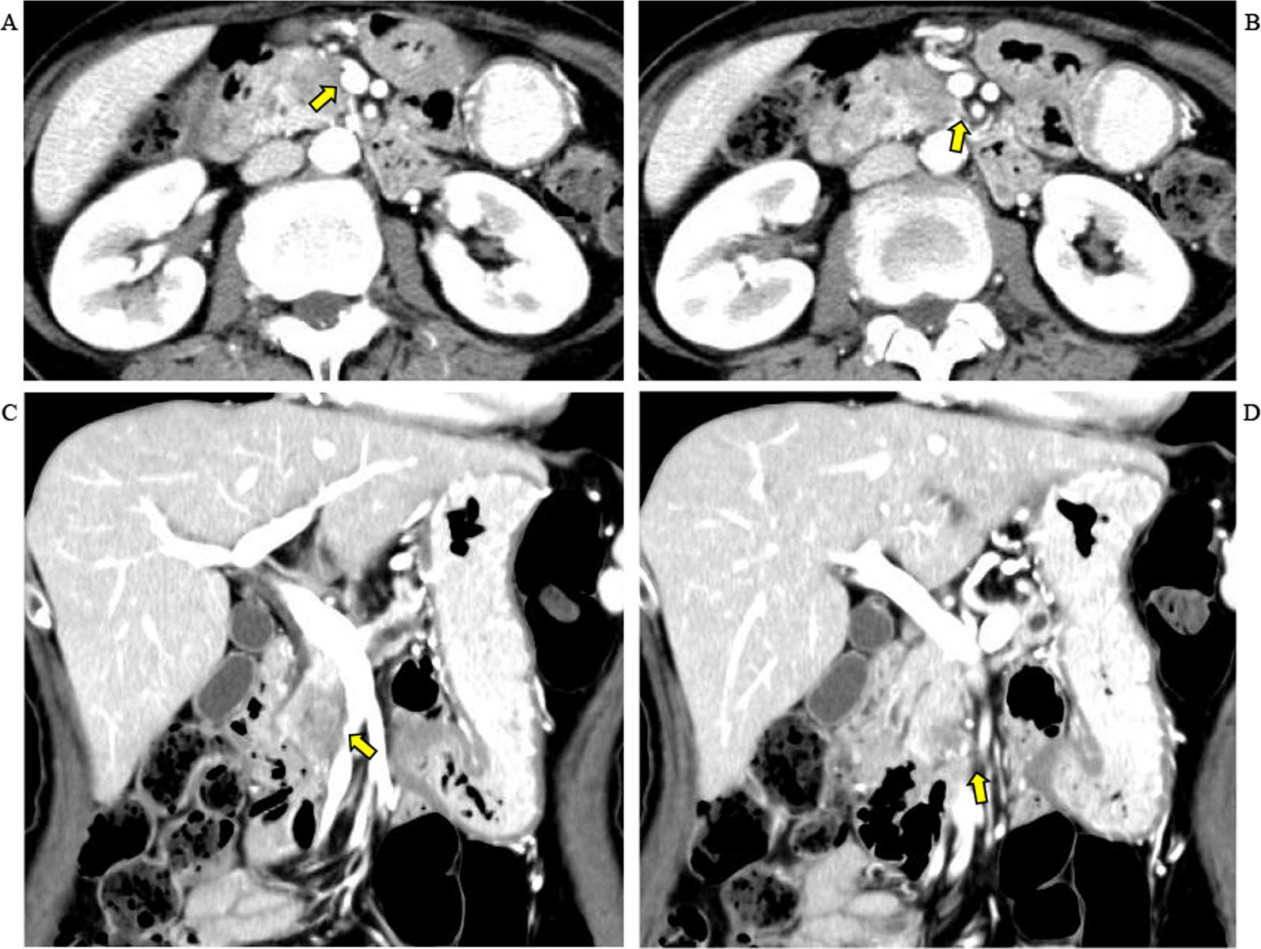
Computed tomographic findings concerning the pancreatic tumor. The pancreatic tumor is in contact with the right side of the superior mesenteric vein (SMV) at the level of confluence of the first jejunal vein tributary, involving less than 180 degrees of the vein’s circumference (**A**). Longitudinal extent of the tumor along the SMV is about 20 mm (**C**). The tumor also has spread along the retropancreatic nerve plexus to a point near the superior mesenteric artery (**B**, **D**)

A 71-year-old woman with a pancreatic tumor involving the head and uncinate process was referred to our hospital with symptoms of back pain, weight loss, and diabetes. According to imaging, the tumor was approximately 30 mm in diameter. The patient was diagnosed with pancreatic duct adenocarcinoma based on findings from an endoscopic ultrasound-guided fine-needle biopsy specimen. The tumor was in contact with the right side of the SMV at the level where it received the first jejunal vein tributary. The tumor attachment to the SMV had a longitudinal extent of about 20 mm but involved less than half of the vein’s circumference, so the tumor was considered resectable. However, extension of tumor along the retropancreatic nerve plexus (RNP) approached the superior mesenteric artery (SMA; Fig. 1), so based upon the results of the PREP-02/JSAP-05 trial [3], 2 cycles of gemcitabine and S-1 were administered before surgery.

Although no radiologically evident changes of tumor status were recognized after chemotherapy, we planned to carry out pancreatoduodenectomy with SMV resection and reconstruction. The first jejunal vein tributary would be resected**,** provided that a vein-clamping test produced no jejunal congestion. Then the segment of the SMV caudal to its confluence with the inferior mesenteric vein (IMV) would be resected in continuity with the pancreatic tumor. Reconstruction of the SMV by simple end-to-end suturing without a venous graft would be difficult, considering that both the splenic vein and the IMV were to be preserved.

**Fig. 2:**
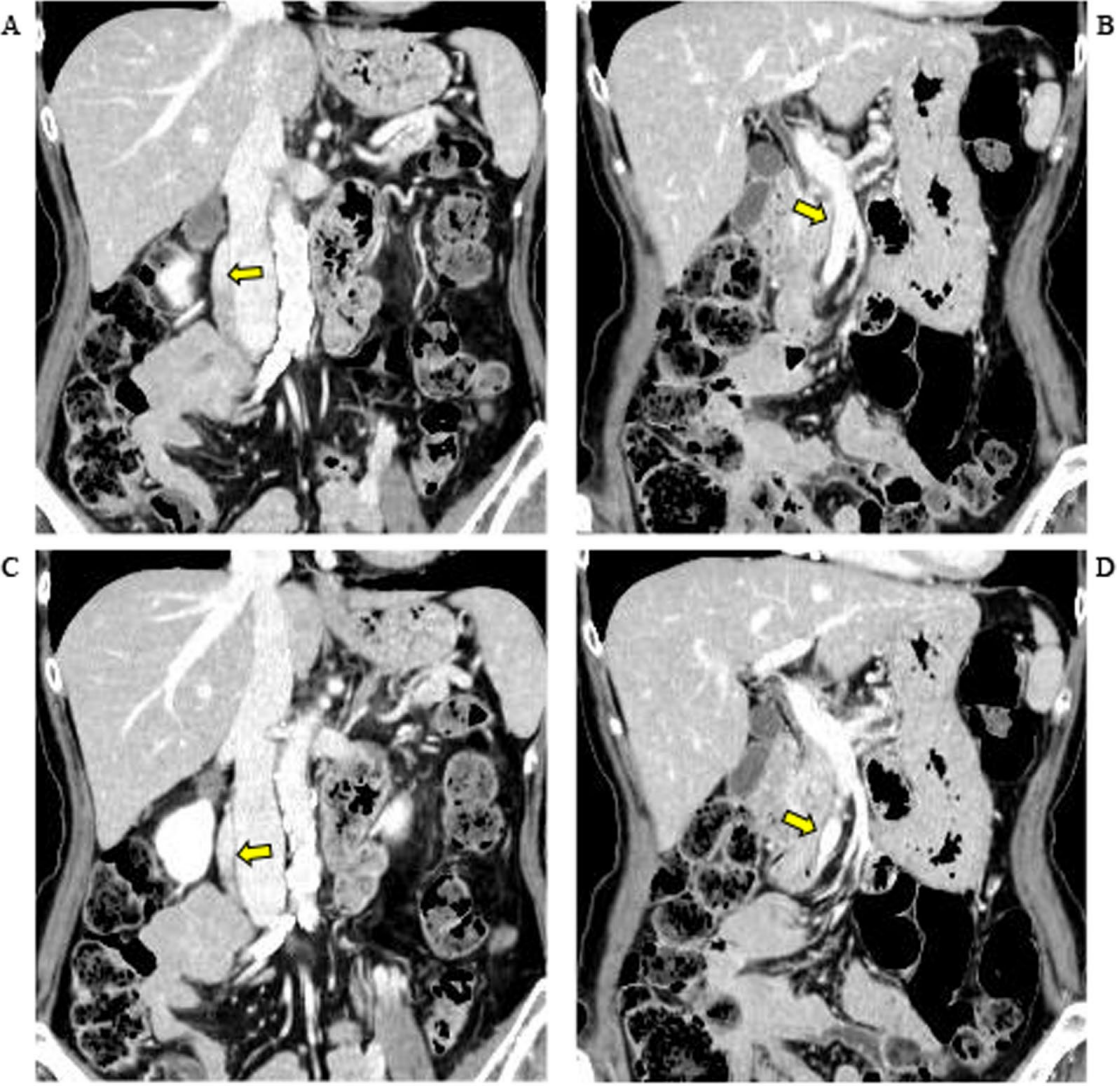
Computed tomographic appearances of the right ovarian vein (**A**, **C**) and the superior mesenteric vein invaded by the tumor (**B**, **D**). The right ovarian vein shows dilation to a diameter of about 10 mm, approximating the dimensions of the portion of the superior mesenteric vein to be resected

One notable finding from computed tomography (CT) before surgery was dilation of the right ovarian vein to a diameter of about 10 mm, similar to the SMV diameter where resection was planned (Fig. 2). Although the patient had no active symptoms of PCS such as chronic pelvic pain, feelings of heaviness, or urinary urgency at the time of surgery, she had experienced severe dysmenorrhea prior to menopause and still had occasional feelings of pelvic heaviness, suggesting that she was at future risk for PCS. We reasoned that use of the dilated right ovarian vein as a graft might prevent or lessen such occurrences while also offering dimensions favorable for grafting.

**Fig. 3:**
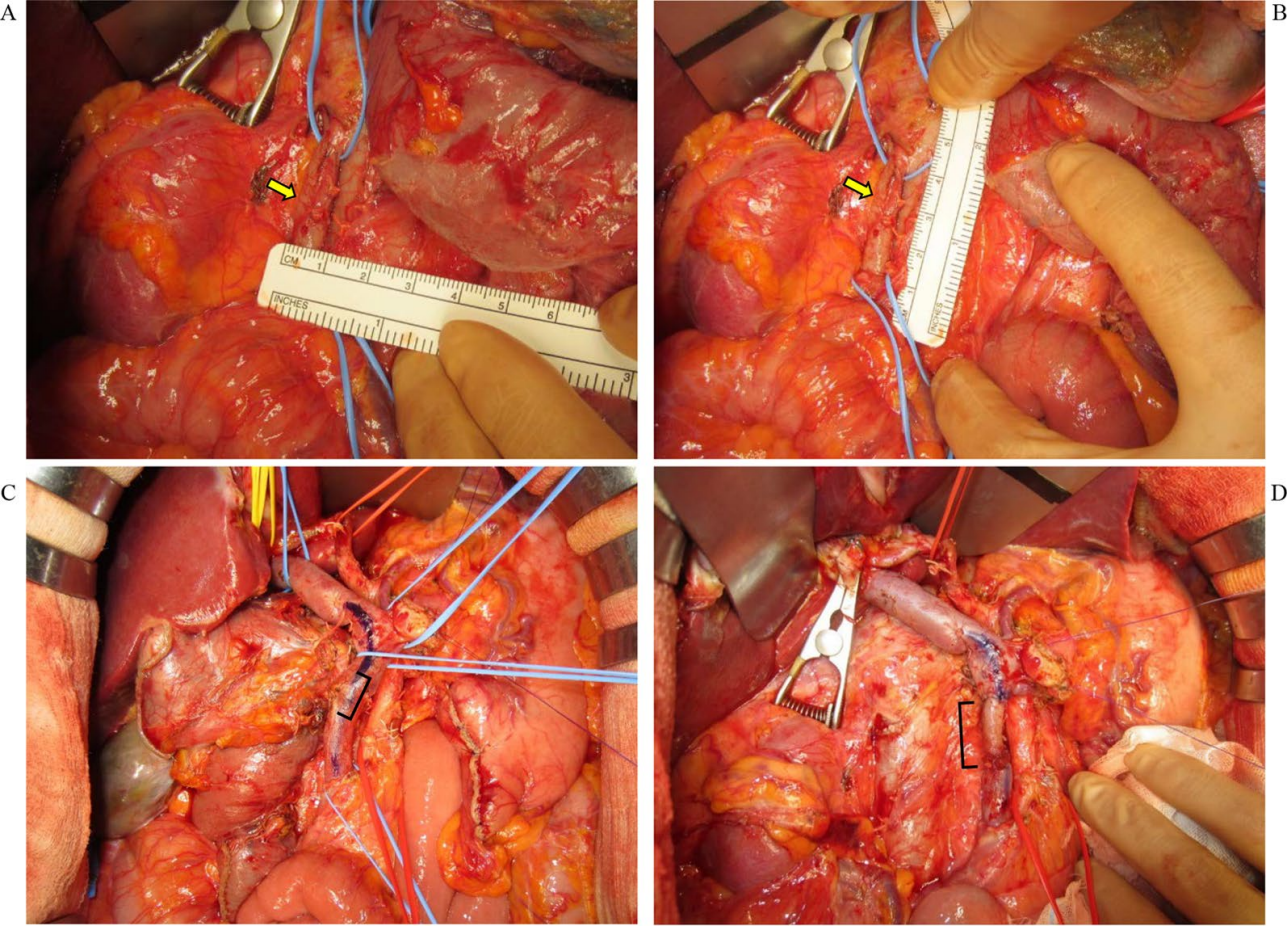
Intraabdominal findings during the operation. **A** and **B** Dilated right ovarian vein to be used for the graft. **C** Superior mesenteric vein invaded by the pancreatic tumor. **D** Superior mesenteric vein after reconstruction using the right ovarian venous graft

No cancer dissemination was grossly evident at laparotomy; subsequent cytologic examination of abdominal lavage fluid detected no malignant cells. Dissection began with liberal application of the Kocher maneuver to the duodenum and firm retraction of the pancreatic head to the left. The peritoneum at the base of the transverse mesocolon was divided to approach the SMA and SMV, as in the mesenteric approach [4]. When no evident jejunal congestion resulted from clamping the first jejunal vein tributary near the confluence of the tributary and the SMV, the tributary was ligated and divided. We performed the retropancreatic nerve plexus (RNP) hanging maneuver as we previously reported [5], making sure that the cut end of the RNP was free of cancer invasion. In brief, the RNP hanging maneuver was carried out as follows. The middle colic artery arising from the SMA and the middle colic vein both were ligated and divided, after which the SMA trunk was followed cranially to its origin. The tape for RNP hanging was placed around the RNP, which was divided together with ligation and division of the inferior pancreatoduodenal artery in the plane determined by the hanging tape prior to any division of the pancreatic parenchyma and gastric antrum. A 3-cm minimum length of the dilated right ovarian vein was removed for use as a venous graft. The tumor was removed together with the portion of the SMV that it had invaded. The SMV was reconstructed with no venous bypass, using the right ovarian vein graft. Obvious valves were not recognized within the patient’s graft after harvesting, so we carried out reconstruction in a standard manner: distal end to distal end and proximal end to proximal end. The duration of reconstruction was 38 min (Fig. 3), while total duration of the operation was 607 min. Intraoperative blood loss was 390 mL. The pathologic diagnosis was invasive ductal carcinoma; the stage according to the classification of pancreatic carcinoma [6] was pT3pN1b (4/40) M0, pStage IIB, with invasion of the SMV (pPV1). R0 resection status was achieved.

**Fig. 4:**
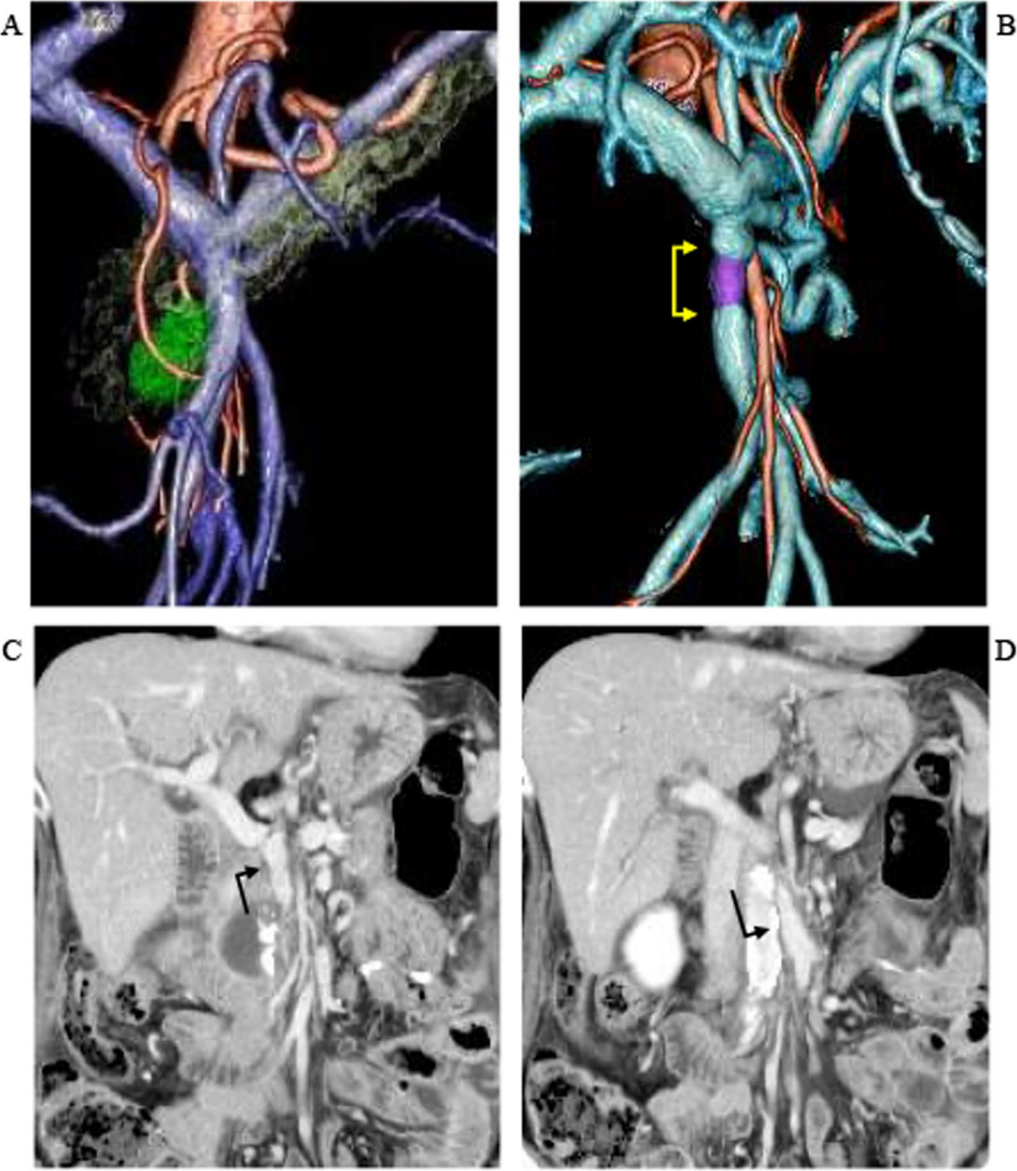
Three-dimensional images of the superior mesenteric vein (SMV) before (**A**) and after (**B**) combined resection and reconstruction. The purple shading represents the ovarian vein graft in place after ligation and division of the first jejunal vein tributary and segmental resection of the SMV followed by SMV reconstruction. **C** and **D** Computed tomographic findings after reconstruction (coronal view)

The postoperative course was uneventful, and the patient was discharged 25 days after surgery. She has finished adjuvant chemotherapy and was free from disease recurrence at 8 months after surgery. PCS symptoms have not occurred. Patency of the venous graft for SMV reconstruction was confirmed by contrast CT carried out with administration of a direct oral anticoagulant 8 months after surgery (Fig. 4).

## Discussion

During pancreatoduodenectomy with venous resection for pancreatic cancer, end-to-end anastomosis is the preferred option for PV or SMV reconstruction. However, when the extent of the PV or SMV resection precludes a simple anastomosis, a variety of vascular grafts can substitute for the resected segment. Because of increased risk of life-threatening thrombotic occlusion, prosthetic grafts are not used except in emergencies where speed is essential or when a suitable autologous vein graft cannot be found. According to the literature, effective replacements for the PV and SMV in elective procedures have included large-caliber vein grafts such as the internal jugular vein, left renal vein, great saphenous vein, external iliac vein, and superficial femoral vein [7]; alternatively, a channel could be fashioned from the falciform ligament [8]. Each potential autologous graft source has advantages and disadvantages. With the internal jugular and lower limb veins, graft harvesting from any segment may be straightforward but preparation of the required additional surgical field distant from the abdomen can be bothersome and time-consuming for the staff as well as a source of difficulty for the patient. The left renal vein may be obtained using the same operative field used for pancreatic surgery, but is sometimes too large for SMV reconstruction. Additionally, many surgeons hesitate to use it unless renal function is optimal. Still another disadvantage of a renal vein graft is that only a relatively short length can be safely harvested. Procurement of the great saphenous vein is largely free of potential complications, but its diameter is too small, requiring spiral reformation prior to grafting [9]; such remodeling is technically demanding and time-consuming. Shortcomings of an external iliac vein graft are congestion and unsightly edema in its territory, as well as pain and pressure sensations upon prolonged standing or walking [10].

Although the left renal vein or the internal jugular vein is usually chosen as a graft for SMV reconstruction at our institution, an ovarian vein graft was used in the present case. Several reports have described SMV reconstruction using gonadal veins [11–13]. Ochiai et al. [11] previously reported a case in which a right ovarian vein was used as a graft during pancreatoduodenectomy, although those authors customized the graft to increase its diameter. In other reports [12, 13] a customized gonadal vein graft was used similarly because the original diameter of the gonadal vein might be insufficient for reconstructing the SMV. Considering that a study of cadaveric ovarian veins showed an average diameter of 3.93 ± 1.11 mm [14], customization of an ovarian vein graft usually would be required for use in SMV reconstruction. In our case, using an ovarian vein showing preexisting dilation eliminated any need for customizing the graft and also was hoped to decrease the likelihood of future re-emergence of pelvic congestion symptoms.

While still in situ, the dilated ovarian vein chosen as a graft in the present patient may have been responsible for her previous symptoms of PCS, a disorder which has a prevalence of 10% to 40% in women over a wide age range. Symptoms of PCS include chronic pelvic pain or feelings of heaviness, dysmenorrhea, dyspareunia, urinary urgency, and perineal or lower limb varices. The underlying anatomic and physiologic abnormalities are ovarian vein dilation and insufficiency [2]. More than half of patients with ovarian vein varices have PCS [15]. The ovarian veins follow a caudal-rostral retroperitoneal course parallel with the spinal column until they drain into the renal vein on the left and into the inferior vena cava on the right, representing only a part of the complex venous network related to the female pelvis. In the study of cadaveric ovarian veins mentioned above with respect to their diameters, mean distances of the termination points of the right and left ovarian veins from the confluences of the right and left renal veins with the IVC were 28.12 ± 7.54 mm and 28.49 ± 5.76 mm, respectively [14].

Importantly, PCS results from a combination of factors [2, 15], and ovarian vein dilation is not the sole etiology. Nonetheless, such dilation contributes significantly to development of PCS as a consequence of vascular congestion reflecting retrograde flow in an incompetent ovarian vein. A previous report proposes an ovarian vein diameter exceeding 8 mm according to contrast CT or magnetic resonance imaging as a criterion for diagnosis of pelvic varices and PCS [16]. While the present patient had no symptoms of PCS at the time of pancreatic surgery, she had a history of severe dysmenorrhea in her premenopausal years and still had occasional feelings of pelvic heaviness.

Hysterectomy formerly was a treatment for PCS, but studies reported residual pain in 33% of patients and recurring symptoms in 20%. This led to a preference for surgical ligation or resection of ovarian veins. Bilateral laparoscopic ligation of ovarian veins has been gaining popularity among gynecologists, but surgical experience with ovarian vein ligation is anecdotal, consisting mainly of a few case studies [1]. A more recent treatment for PCS is percutaneous embolization; a high percentage of patients have reported symptom improvement [2]. At this writing, the patient has noted no recurrence of pelvic heaviness since the operation, but further observation remains necessary to establish efficacy of her ovarian vein resection in preventing symptom recurrence.

Diameters of our patient’s dilated right ovarian vein and the portion of the SMV to be resected and reconstructed were similar, so we chose this vein for the graft even though previous symptoms suggestive of PCS were no longer evident. Use of this vein eliminated any need for an additional surgical field or customizing a graft to increase its diameter. Future pelvic congestive symptoms also should be less likely in this patient. Good graft patency has been maintained postoperatively, and the patient has completed adjuvant chemotherapy.

## Conclusion

In female patients requiring pancreatoduodenectomy with segmental SMV resection who have ovarian vein dilation predisposing to PCS, this dilated vein may be an advantageous choice for use as a graft to reconstruct the SMV.

## Data Availability

Not applicable.

## Abbreviations

CT: Computed tomography
PCS: Pelvic congestion syndrome
PV: Portal vein
RNP: Retropancreatic nerve plexus
SMV: Superior mesenteric vein
